# Differential Effect of SARS-CoV-2 Spike Glycoprotein 1 on Human Bronchial and Alveolar Lung Mucosa Models: Implications for Pathogenicity

**DOI:** 10.3390/v13122537

**Published:** 2021-12-17

**Authors:** Mizanur Rahman, Martin Irmler, Sandeep Keshavan, Micol Introna, Johannes Beckers, Lena Palmberg, Gunnar Johanson, Koustav Ganguly, Swapna Upadhyay

**Affiliations:** 1 Unit of Integrative Toxicology, Institute of Environmental Medicine, Karolinska Institutet, 17 177 Stockholm, Sweden; mizanur.rahman@ki.se (M.R.); micolintr@gmail.com (M.I.); lena.palmberg@ki.se (L.P.); gunnar.johanson@ki.se (G.J.); 2 Institute of Experimental Genetics, Helmholtz Zentrum München GmbH, 85764 Neuherberg, Germany; martin.irmler@helmholtz-muenchen.de (M.I.); beckers@helmholtz-muenchen.de (J.B.); 3 Adolphe Merkle Institute, University of Fribourg, Chemin des Verdiers 4, 1700 Fribourg, Switzerland; sandeep.keshavan@unifr.ch; 4 German Center for Diabetes Research (DZD e.V.), 85764 Neuherberg, Germany; 5 Chair of Experimental Genetics, Technical University of Munich, 85354 Freising, Germany

**Keywords:** COVID-19, coronavirus, SARS-CoV-2, spike protein, SARS (severe acute respiratory syndrome), MERS (middle east respiratory syndrome), lung, pulmonary, fibrosis

## Abstract

Background: The SARS-CoV-2 spike protein mediates attachment of the virus to the host cell receptor and fusion between the virus and the cell membrane. The S1 subunit of the spike glycoprotein (S1 protein) contains the angiotensin converting enzyme 2 (ACE2) receptor binding domain. The SARS-CoV-2 variants of concern contain mutations in the S1 subunit. The spike protein is the primary target of neutralizing antibodies generated following infection, and constitutes the viral component of mRNA-based COVID-19 vaccines. Methods: Therefore, in this work we assessed the effect of exposure (24 h) to 10 nM SARS-CoV-2 recombinant S1 protein on physiologically relevant human bronchial (bro) and alveolar (alv) lung mucosa models cultured at air–liquid interface (ALI) (*n* = 6 per exposure condition). Corresponding sham exposed samples served as a control. The bro-ALI model was developed using primary bronchial epithelial cells and the alv-ALI model using representative type II pneumocytes (NCI-H441). Results: Exposure to S1 protein induced the surface expression of ACE2, toll like receptor (TLR) 2, and TLR4 in both bro-ALI and alv-ALI models. Transcript expression analysis identified 117 (bro-ALI) and 97 (alv-ALI) differentially regulated genes (*p* ≤ 0.01). Pathway analysis revealed enrichment of canonical pathways such as interferon (IFN) signaling, influenza, coronavirus, and anti-viral response in the bro-ALI. Secreted levels of interleukin (IL) 4 and IL12 were significantly (*p* < 0.05) increased, whereas IL6 decreased in the bro-ALI. In the case of alv-ALI, enriched terms involving p53, APRIL (a proliferation-inducing ligand) tight junction, integrin kinase, and IL1 signaling were identified. These terms are associated with lung fibrosis. Further, significantly (*p* < 0.05) increased levels of secreted pro-inflammatory cytokines IFNγ, IL1ꞵ, IL2, IL4, IL6, IL8, IL10, IL13, and tumor necrosis factor alpha were detected in alv-ALI, whereas IL12 was decreased. Altered levels of these cytokines are also associated with lung fibrotic response. Conclusions: In conclusion, we observed a typical anti-viral response in the bronchial model and a pro-fibrotic response in the alveolar model. The bro-ALI and alv-ALI models may serve as an easy and robust platform for assessing the pathogenicity of SARS-CoV-2 variants of concern at different lung regions.

## 1. Introduction

The spike protein of the severe acute respiratory syndrome coronavirus 2 (SARS-CoV-2), responsible for causing the coronavirus disease-2019 (COVID-19), mediates attachment of the virus to host cell-surface receptor and fusion between virus and cell membrane [1]. The spike protein consists of two subunits: S1 (containing the angiotensin converting enzyme 2 (ACE2) receptor-binding domain) and S2 (responsible for the fusion process) [2]. The SARS-CoV-2 spike protein is the primary target of neutralizing antibodies generated following infection. It also constitutes the viral component of both mRNA and adenovirus-based vaccines for COVID-19. Therefore, spike proteins and mutations that can affect the antigenicity of spike proteins are of particular interest.

At the time of writing this article, five variants of concern of SARS-CoV-2, namely alpha, beta, gamma, delta, and omicron have been declared by the World Health Organization based on evidence of impact on transmissibility, immunity, and severity [3,4]. The variants of concern have mutations in the S1 subunit [3,4]. Expression of ACE2 in the lung have been detected in the ciliated airway epithelial cells and the motile cilia of cells lining the nasal turbinate, sinus, trachea, and bronchus, as well as in type II pneumocytes of the alveoli. [5,6]. Analysis of nasopharyngeal swabs, bronchial brushes, and bronchoalveolar lavage showed an upregulation of ACE2 mRNA in COVID-19 patients compared to healthy subjects [7,8]. Examination of lungs of deceased COVID-19 patients compared to uninfected age-matched control subjects revealed greater number of ACE2-positive alveolar epithelial cells [8,9]. In this context, the plausible role of using bronchoalveolar lavage fluid among patients with a negative COVID-19 from nasopharyngeal swab to establish a different diagnosis may be considered by performing additional molecular analysis [10]. One study reported high lymphocytic infiltration in the bronchoalveolar lavage fluid during COVID-19 pneumonia [11]. Therefore, data on alveolar cellularity and corresponding molecular analysis of bronchoalveolar lavage fluid may open up new immunomodulatory treatment strategies for COVID-19 [11].

Toll-like receptors (TLRs) are innate immune receptors present on the cell surface that recognize pathogen-associated molecular patterns including viral proteins. TLRs play an important role in innate immunity by triggering the production of type I interferons and proinflammatory cytokines. Both TLR2 and TLR4 have been implicated in SARS-CoV-2 infection [12,13,14,15]. Based on the similarity of SARS-CoV-2 with other coronaviruses, it has been hypothesized that pathways involving nuclear factor kappa-light-chain-enhancer of activated B cells (NF-kB), transforming growth factor-β, cytokine regulation, p53, epidermal growth factor receptor, c-Jun *n*-terminal protein kinases, p38 mitogen-activated protein kinases, extracellular signal-regulated kinase, tumor necrosis factor-alpha (TNFα), and interferon (IFN) signaling may be affected in COVID-19 [16]. Recent studies reported that the recombinant SARS-CoV-2 spike glycoprotein S1 subunit alone elicits cell signaling in primary human pulmonary artery smooth muscle cells and human pulmonary artery endothelial cells [2,17]. The findings are consistent with the detection of pulmonary vascular wall thickening, a key feature of pulmonary arterial hypertension, indicating pulmonary vascular remodeling [2,17] in lungs of deceased COVID-19 patients. It has become increasingly evident that COVID-19 patients develop features of interstitial pulmonary fibrosis (IPF) [18]. The characteristics of IPF is excessive extracellular matrix deposition within the lung interstitium, leading to the destruction of normal parenchymal structure and a corresponding loss of pulmonary function. The meta-analysis study performed by Ouyang et al. [19] that included 135,263 COVID-19 patients from 15 cohorts concluded that pre-existing interstitial lung disease is associated with higher severity and mortality of COVID-19. The most common short-term post-COVID-19 fibrotic patterns included ground glass opacities and linear bands, bronchiectasis/bronchiolectasis, and loss of volume in the bilateral posterior lung segments. However, it remains to be seen if the COVID-19 related fibrotic changes are reversible, progressive, or permanent [20].

Therefore, it is important to explore the early molecular events of COVID-19-related fibrotic changes in the lung so as to develop therapeutic strategies to counter long-term deleterious effects.

In this work, we exposed physiologically relevant bronchial (bro) and alveolar (alv) lung mucosa models developed at air–liquid interface (ALI) with the full length recombinant spike glycoprotein S1 domain from SARS-CoV-2, Wuhan-Hu-1, to assess transcriptomic alterations and pro-inflammatory cytokine secretion. The bronchial mucosa models (bro-ALI) were developed using human primary bronchial epithelial cells (PBEC) and the alveolar model (alv-ALI) was developed using representative human type II pneumocytes. Our study identified differential effects of the SARS-CoV-2 S1 subunit on two different lung regions that may have implications to assess the pathogenicity and immune escape of the variants of concern, as well as long-term consequence of vaccines.

## 2. Material and Methods

### 2.1. Recombinant Spike Glycoprotein S1

Spike glycoprotein S1 domain from SARS-related coronavirus-2, Wuhan-Hu-1, with C-terminal histidine tag, recombinant from HEK293 cells (S1 protein) (Catalog # NR-53798), was obtained through BEI resources, NIAD, NIH (Manassas, VA, US). The NR-53798 lacks the signal sequence, contains 670 residues of the SARS-CoV-2 spike glycoprotein (aa: V16–R685) and features a C-terminal poly-histidine tag. The predicted molecular weight of NR-53798 is 76,500 Da.

### 2.2. Bronchial and Alveolar Lung Mucosal Models:

#### 2.2.1. Bronchial

The bro-ALI model was developed using PBEC harvested from healthy bronchial tissue obtained from a donor in connection with lobectomy following written and informed consent, and approval by the Swedish Ethical Review Authority (Institutional ethic committee reference number 99–357, approved on 10 January 2000). The detailed protocol and details of cellular differentiation (club cells, goblet cells, basal cells, ciliated cells, etc.) of the bro-ALI model have been described previously [21] and have been used in several studies [21,22,23]. All experiments and methods were carried out in accordance with relevant guidelines and regulations.

#### 2.2.2. Alveolar

The alv-ALI model was developed using NCI-H441 (ATCC HTB-174) cell line, known to express constitutively the mRNA and protein of the major surfactant apo-protein. NCI-H441 cells were co-cultured with HULEC-5a (ATCC CRL-3244), representative of human lung microvascular endothelial cells for this purpose. Details of the development of alv-ALI model and its characteristics have been described recently [24]. The characterization included light-, confocal-, transmission electron microscopy, and transepithelial electrical resistance measurement of the differentiated H441 at the ALI condition. Morphological characterization of the alveolar mucosa model demonstrated the presence of tight junction protein 1, lamellar bodies, surfactant protein C, microvilli, lipid bodies, desmosome, and tight junctions [24].

### 2.3. Exposure to Spike Protein

Of the S1 protein, 10 nM (in 80 µL cell culture medium) was added on the apical surface of both bro-ALI and alv-ALI and incubated for 24 h. The exposure dose [25] was determined following a dose gradient study using 5, 10, and 20 nM S1 protein and corresponding assessment of cytotoxicity, surface expression of ACE2, TLR2, and TLR4 by flow cytometry. Exposed samples were compared to the corresponding sham (cell culture media without S1 protein). Six (*n* = 6 per exposure condition) replicates randomly distributed in the plates and experiments performed on different days were used for both bro-ALI (developed from one donor) and alv-ALI (developed from different cell vials) for flow cytometry, transcriptomic, and cytokine secretion assays.

### 2.4. Cytotoxicity Assessment

Lactate dehydrogenase (LDH) assay and propidium iodide (PI) staining: To access cell viability (based on membrane integrity) in both bro-ALI and alv-ALI models, LDH (Thermo Fisher scientific Rockford, IL, USA, catalog # 88953) and propidium iodide assay (BD bioscience, San Jose, CA, USA, catalog # 556463) were performed according to manufacturer’s instructions. The colorimetric LDH assay was measured using a BioTek 800 TS absorbance reader (Santa Clara, CA, USA) and PI assay was performed using flow cytometry (BD LSRFortessa cell analyzer, BD bioscience, San Jose, CA, USA) following 24 h post-exposure with 5, 10, and 20 nM S1 protein (*n* = 3 per exposure condition). The flow cytometric data was analyzed using FlowJo software-7.6.1 (BD bioscience, San Jose, CA, USA). Data are presented as percentage positive PI cells and interquartile range. LDH assay is shown as median of absorbance (450 nm) and interquartile range.

### 2.5. Surface Expression of ACE2, TLR2, and TLR4

Following 6 h exposure of both bro-ALI and alv-ALI with S1 protein, cells were trypsinzed and collected by centrifugation (1500 rpm for 10 min). This was followed by washing (twice) with PBS. The cells were resuspended with PBS (100 µL) and then incubated with antibodies against ACE2 (Biotechne, Abingdon, UK, catalog # FAB9332G), TLR2 (BD bioscience, San Jose, CA, USA, catalog # 565350), and TLR4 (BD bioscience, San Jose, CA, USA, catalog # 564404) for 30 min on ice (according to manufacturer’s instruction). Finally, the cells were washed thrice and re-suspended in PBS. The surface expression of ACE2, TLR2 and TLR4 were measured using flow cytometry (BD LSRFortessa cell analyzer, BD bioscience, San Jose, CA, USA). The flow cytometric data was analyzed using FlowJo software-7.6.1 (BD bioscience, San Jose, CA, USA). Data are presented as MFI and interquartile range.

### 2.6. Confocal Microscopy of ACE2 Expression

Both bro-ALI (*n* = 6) and alv-ALI (*n* = 6) models exposed to 10 nM S1 protein for 6 h were fixed by adding 1 mL of 4 % formalin of paraformaldehyde on both (apical and basal) sides of the inserts and incubated for 30 min. After that, model membranes were washed twice using PBS and then membranes were incubated with blocking buffer (PBS + 0.1% Triton X + goat serum) for maximum 15 min. Then the membranes were stained with primary antibodies against ACE2 primary antibody (rabbit anti-human ACE2, dilution 1:1000, Thermo Fisher, Stockholm, Sweden, Catalog # PA520046) overnight at 4 °C. On day 2, membranes were incubated with secondary antibodies: Alexa Fluor 488-conjugated goat anti-rabbit IgG (1:500; Abcam, Cambridge, UK, catalog # ab150077), Finally, the membranes were mounted on microscope slides with 4′,6-diamidino-2-phenylindole (DAPI; Abcam, Cambridge, UK, catalog # ab104139). Images were captured and visualized using a LSM700 confocal microscope (Zeiss, Oberkochen, Baden-Württemberg, Germany). Corresponding sham exposed samples were used as controls.

### 2.7. Transcriptomic Analysis

Transcriptomic analysis was performed using the UPX 3′ RNA sequencing technology (Qiagen Genomic Services, Hilden, Germany). To determine the differentially exposed genes following 24 h of exposure to 10 nm S1 protein in both bro-ALI (*n* = 6) and alv-ALI (*n* = 6) compared to the corresponding sham, cells were collected in Qiagen RLT buffer (Qiagen, Hilden, Germany, catalog # 74104), snap frozen, and dispatched in dry ice to the service laboratory as per the service provider’s instructions. Transcriptomic analysis workflow is briefly described in the Supplementary Materials. For alv-ALI, the apical layer containing type II pneumocytes were collected. All steps in the process passed the quality check of the service provider. A raw *p* value ≤ 0.01 was set to select differentially expressed genes. Heatmaps showing the top 20 upregulated and 20 downregulated genes were generated in R [26]. Ensembl genes without gene symbol annotation were omitted from the heatmaps. RNAseq data is deposited at the Gene Expression Omnibus database at NCBI (GSE185657; https://www.ncbi.nlm.nih.gov/geo/; accessed on 29 November 2021.).

Pathway and enrichment analysis: For the biological interpretation of the observed gene regulation, pathway and gene ontology term enrichment analyses was performed using the QIAGEN’s Ingenuity Pathway Analysis software (IPA^®^, QIAGEN Redwood City, www.qiagen.com/ingenuity, version: 65367011; accessed on 10 October 2021.) and by g:profiler (version e104_eg51 _p15_3922dba, https://biit.cs.ut.ee/gprofiler/gost, accessed on 10 October 2021) [27]. Significant terms were selected using Fisher’s Exact Test *p*-values (IPA) or g:SCS < 0.05 (g:profiler).

### 2.8. Secreted Cytokine Concentration

Concentrations of IFNγ, interleukin (IL) IL1B, IL2, IL4, IL6, IL8, IL10, IL12, IL13, and tumor necrosis factor alpha (TNFα) were measured in the basal media of bro-ALI (*n* = 6) and alv-ALI (*n* = 6) following 24 h post-exposure with 10 nM S1 protein and compared to the corresponding sham. IL8 was measured using ELISA (R&D Systems, Minneapolis, MN, USA, Catalog # DY208) while the remaining cytokines were measured using the V-plex immunoassay platform of Meso Scale Discovery Inc (Rockville, MD, USA) at the Clinical Biomarkers Facility, Science for Life Laboratory, Uppsala University, Sweden. Limits of detection of the kits is provided in the Supplementary Table S1.

### 2.9. Statistics

The results (flow cytometry, protein concentration) are expressed as median and interquartile ranges (25th–75th percentiles) followed by non-parametric statistical analysis (Mann–Whitney *U* test). All the data were analyzed using the GraphPad Prism (8.3.0) software (LaJolla, CA, US). A *p* value < 0.05 was considered as significant. Statistics relevant to RNA sequencing and pathway analysis are mentioned in the respective sections.

## 3. Results

**Figure 1:**
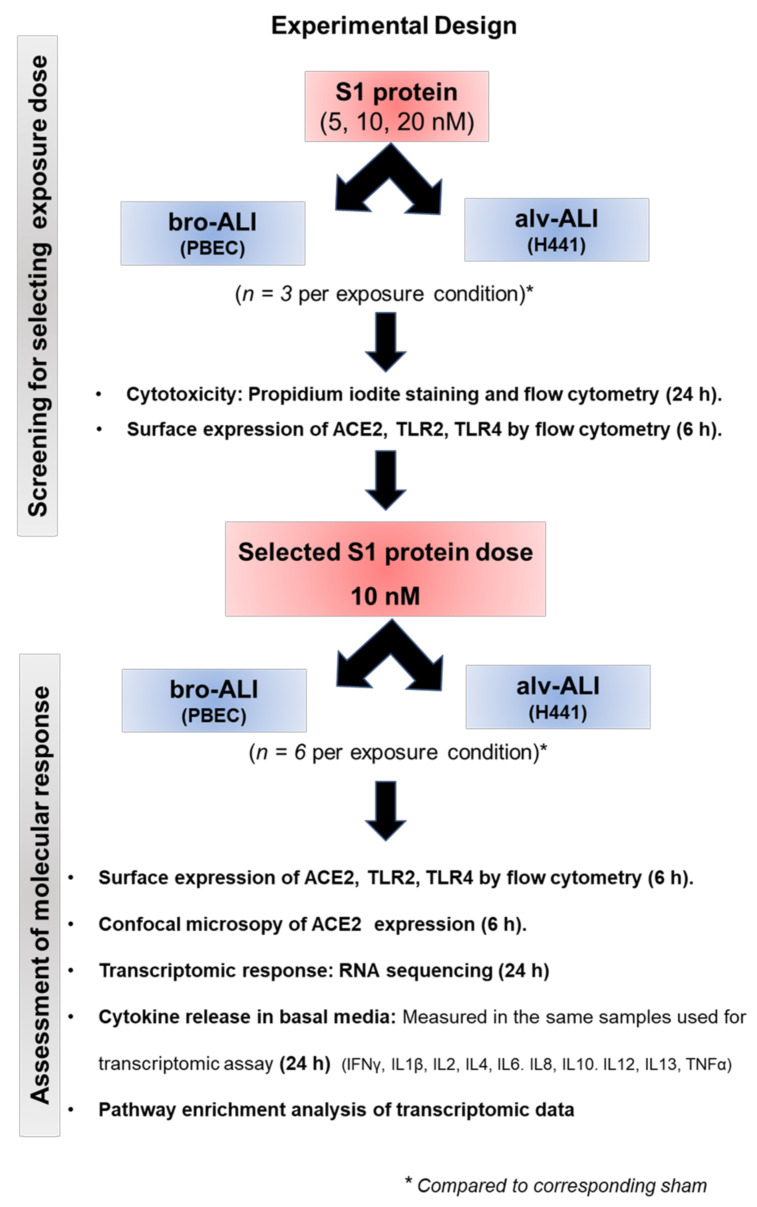
Schematic presentation of the overall experimental design. ACE2: angiotensin converting enzyme 2; ALI: air–liquid interface; alv-ALI: alveolar lung mucosa model developed at ALI; bro-ALI: bronchial lung mucosa model developed at ALI; h: hour; H441: NCI-H441 cell line as representative human type II pneumocytes; IFN: interferon; IL: interleukin; PBEC: human primary bronchial epithelial cells; S1 protein: spike glycoprotein S1 domain from SARS-related coronavirus-2; TLR: toll-like receptors; TNF: tumor necrosis factor.

The overall experimental design is shown in Figure 1. None of the doses (5, 10, 20 nM) used for screening were cytotoxic (Supplementary Figure S1) and the final exposure dose (10 nM) were selected based on the ACE2, TLR2, and TLR4 expression (Supplementary Figures S2 and S3).

### 3.1. Increased ACE2, TLR2, and TLR4 Surface Expression

**Figure 2:**
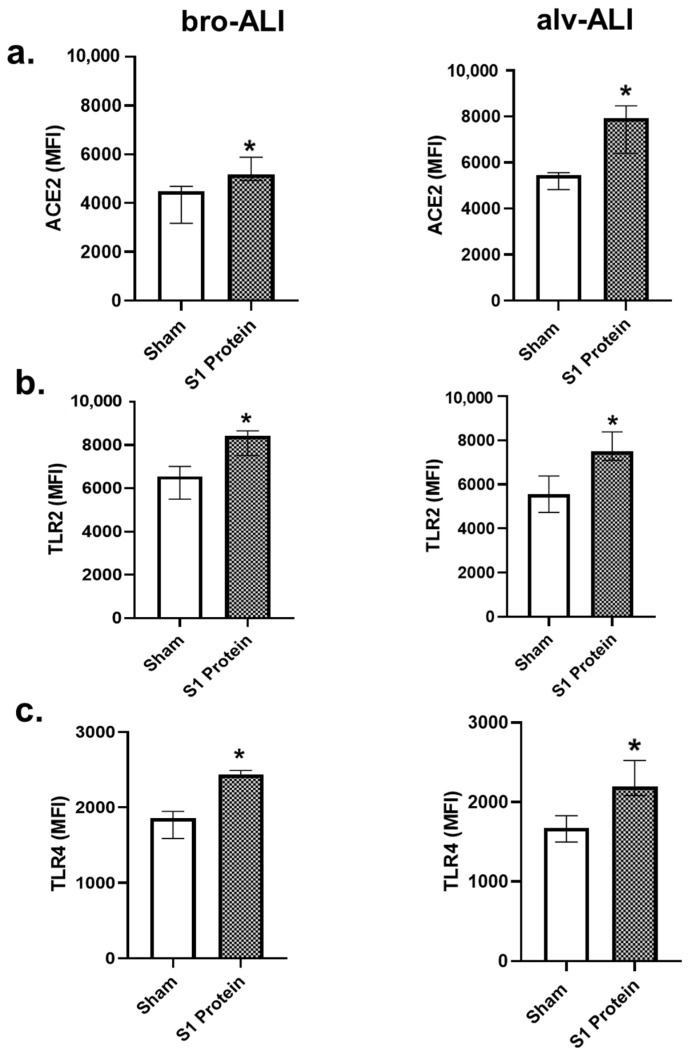
Increased surface expression of angiotensin converting enzyme 2 (ACE2) (**a**), toll-like receptor 2 (TLR2) (**b**), and TLR4 (**c**) in the bronchial (bro-ALI) and alveolar (alv-ALI) mucosa model developed at air–liquid interface. Both bro-ALI and alv-ALI were exposed to 10 nM recombinant SARS-CoV-2 spike glycoprotein S1 (S1 protein) for 6 h and compared to the corresponding sham. ACE2 (**a**), TLR2 (**b**), and TLR4 (**c**) were measured by flow cytometry and data are presented as median fluorescent intensity (MFI) and interquartile range. *n* = 6 per exposure condition; * significance: *p* < 0.05 (Mann–Whitney *U* test).

**Figure 3:**
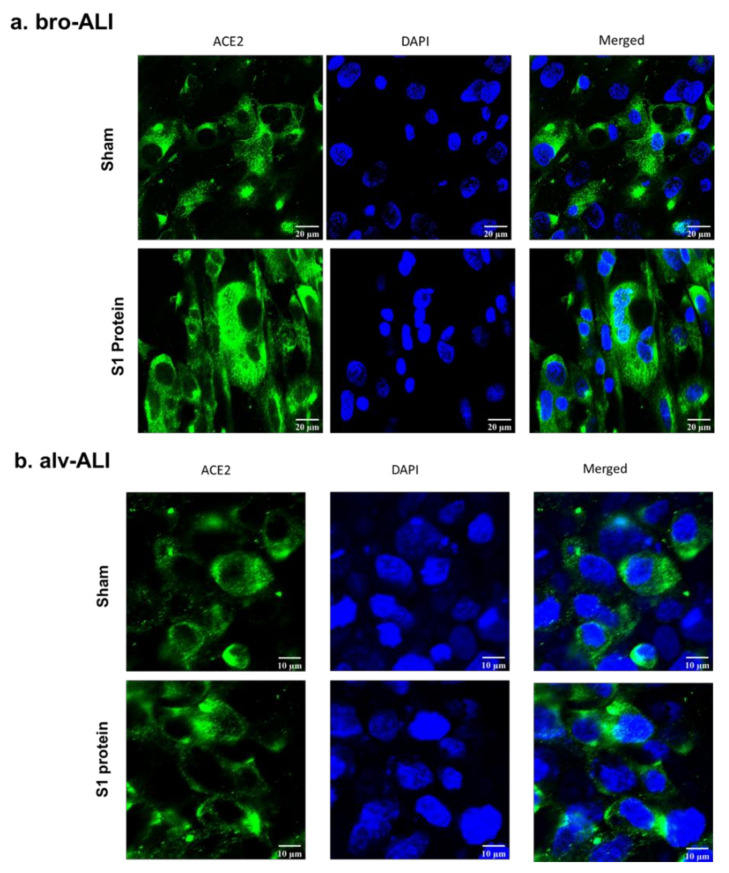
Confocal microscopy of angiotensin converting enzyme 2 (ACE2) expression in the bronchial (bro-ALI: (**a**) and alveolar (alv-ALI: (**b**) mucosa model developed at air–liquid interface. Both bro-ALI and alv-ALI were exposed to 10 nM recombinant SARS-CoV-2 spike glycoprotein S1 (S1 protein) for 6 h and compared to the sham (representative picture of three independent observations). Scale bar bro-ALI: 20 µm; scale bar alv-ALI: 10 µm.

Significantly (*p* < 0.05) increased surface expression of ACE2, TLR2, and TLR4 was detected in both the bro-ALI and alv-ALI (Figure 2a–c; Supplementary Table S2) models exposed to S1 protein. Increased expression of ACE2 in both bro-ALI (Figure 3a) and alv-ALI (Figure 3b) was also detected by confocal microscopy.

### 3.2. Transcriptomic Response in bro-ALI

**Figure 4:**
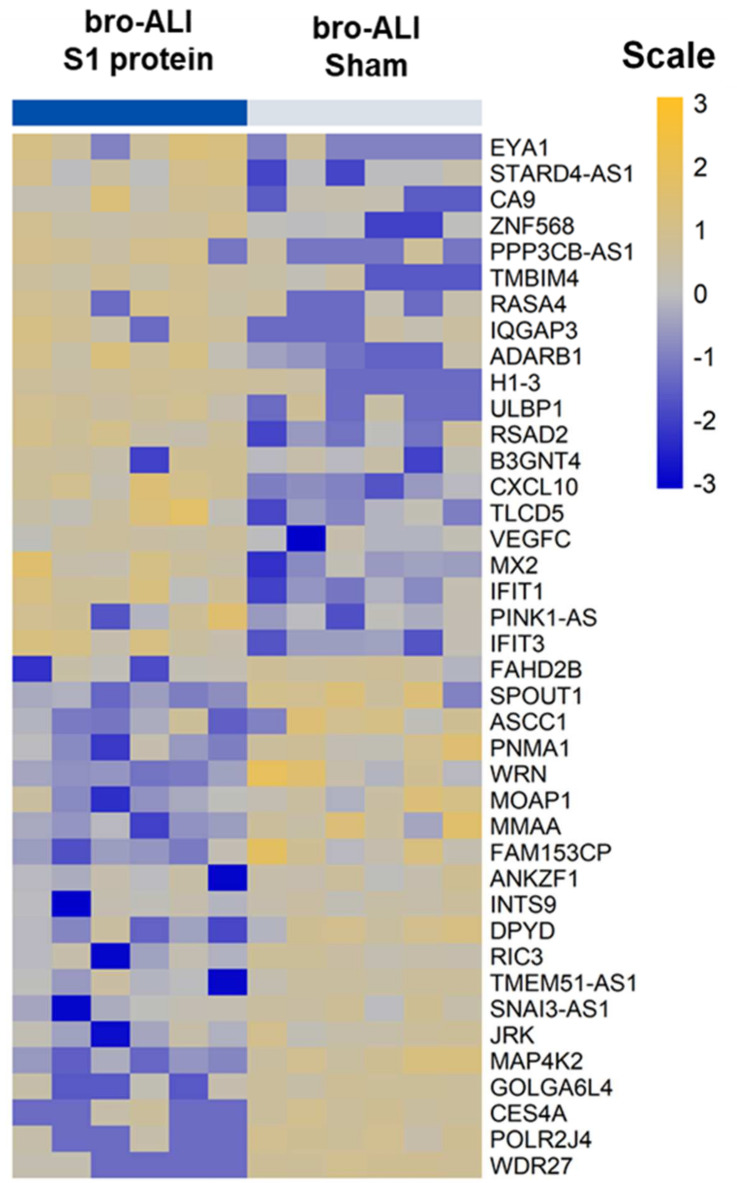
Heatmap of top 20 upregulated and 20 downregulated genes in the bronchial mucosa model developed at air–liquid interface (bro-ALI). bro-ALI was exposed to 10 nM recombinant SARS-CoV-2 spike glycoprotein S1 (S1 protein) for 24 h (h) and compared to the corresponding sham. *n* = 6 per exposure condition; significantly (raw *p* < 0.01) regulated genes with the highest fold changes are shown. Genes were ordered by fold-change (S1 protein vs. sham) and relative gene expression values are shown across samples (z-scales to mean expression per row).

**Figure 5:**
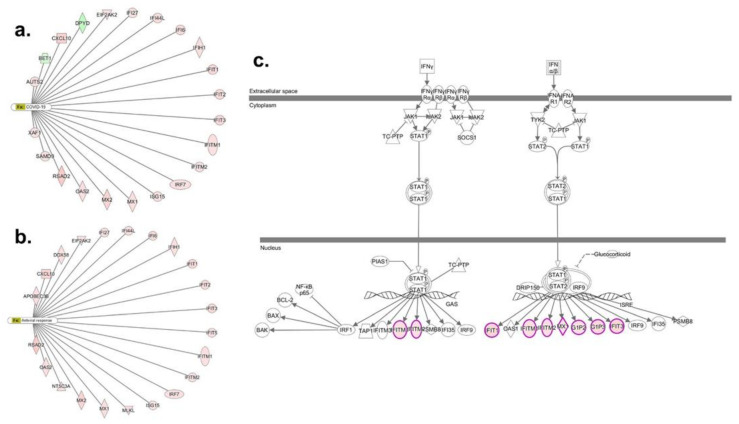
Ingenuity pathway analysis of genes regulated in the bronchial mucosa model developed at air–liquid interface (bro-ALI). bro-ALI was exposed to 10 nM recombinant SARS-CoV-2 spike glycoprotein S1 for 24 h. Genes associated with significantly enriched functions and disease terms (**a**) COVID-19; (**b**) anti-viral response; and (**c**) interferon signaling. Significantly regulated genes are highlighted in red/green for up- or downregulation. Legend for network shapes is provided in Supplementary Figure S4.

A total of 117 genes were differentially regulated (77 upregulated and 40 down regulated; *n* = 6; *p* < 0.01) in the bro-ALI post 24 h exposure to S1 protein compared to sham (Supplementary Table S3). Figure 4 shows the heat map of the top 20 upregulated and 20 downregulated genes in bro-ALI. The significantly enriched canonical pathways generated using the 117 differentially regulated genes in bro-ALI are given in Table 1. An over-representation of the viral response including coronavirus and COVID-19 (Figure 5a), antiviral response (Figure 5b), and interferon signaling (Figure 5c) was observed for bro-ALI in the pathway enrichment analysis. Similar results were also obtained by Gene Ontology term enrichment analysis.

**Figure:**
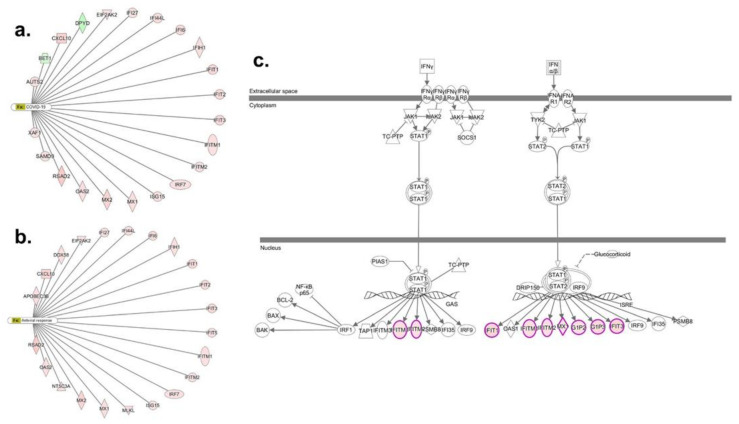


### 3.3. Secreted Cytokines in bro-ALI

**Figure 6:**
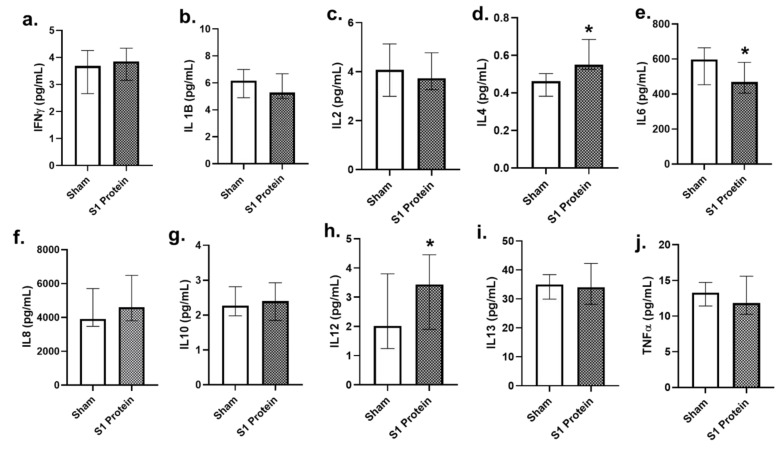
Concentration of secreted proinflammatory cytokines in the basal media of bronchial mucosa model developed at air–liquid interface (bro-ALI). bro-ALI was exposed to 10 nM recombinant SARS-CoV-2 spike glycoprotein S1 (S1 protein) for 24 h and compared to the corresponding sham (**a**–**j**). IFNγ: interferon gamma; IL: interleukin; TNFα: tumor necrosis factor alpha. *n* = 6 per exposure condition; * Significance: *p* < 0.05 (Mann–Whitney *U* test).

Among the 10 pro-inflammatory cytokines assessed (Figure 6), concentrations of IL4 and IL12 (Figure 6d,h) were significantly increased (*p* < 0.05; *n* = 6), whereas that of IL6 (Figure 6e) significantly decreased in the bro-ALI at 24 h post S1 protein exposure, compared to sham.

### 3.4. Transcriptomic Response in alv-ALI

**Figure 7:**
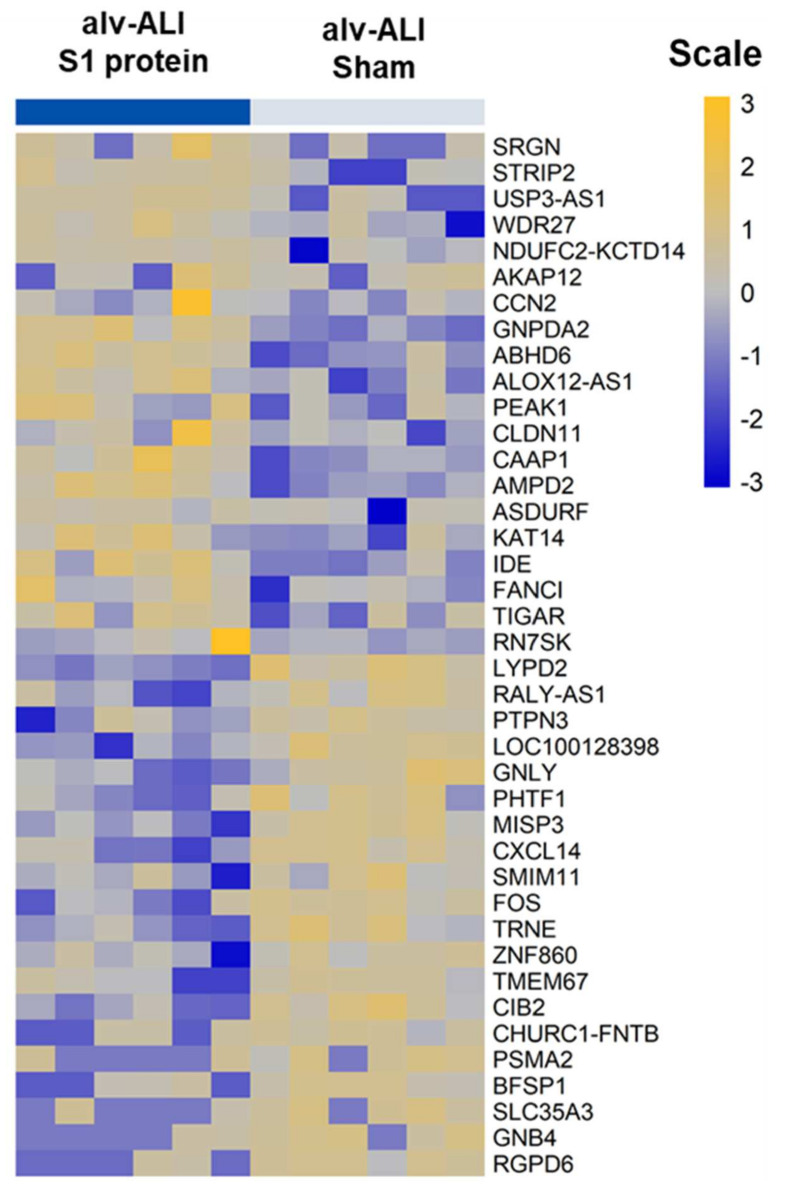
Heatmap of top 20 upregulated and 20 downregulated genes in the alveolar mucosa model developed at air–liquid interface (alv-ALI). alv-ALI was exposed to 10 nM recombinant SARS-CoV-2 spike glycoprotein S1 (S1 protein) for 24 h and compared to the corresponding sham. *n* = 6 per exposure condition; significantly (raw *p* < 0.01) regulated genes with the highest fold changes are shown. Genes were ordered by fold-change (S1 protein vs. sham) and relative gene expression values are shown across samples (z-scales to mean expression per row).

A total of 97 genes were differentially regulated (47 upregulated and 50 down regulated; *n* = 6; *p* < 0.01) in the alv-ALI post 24 h exposure to S1 protein compared to sham (Supplementary Table S4). Figure 7 shows a heat map of the top 20 upregulated and 20 downregulated genes in alv-ALI. The significantly enriched canonical pathways based on the 97 differentially regulated genes in alv-ALI are given in Table 1. These include p53 signaling, a proliferation-inducing ligand (APRIL)-mediated signaling, tight junction signaling, integrin-linked kinase (ILK) signaling, agranulocyte adhesion and diapedesis, and IL1 signaling.

**Figure:**
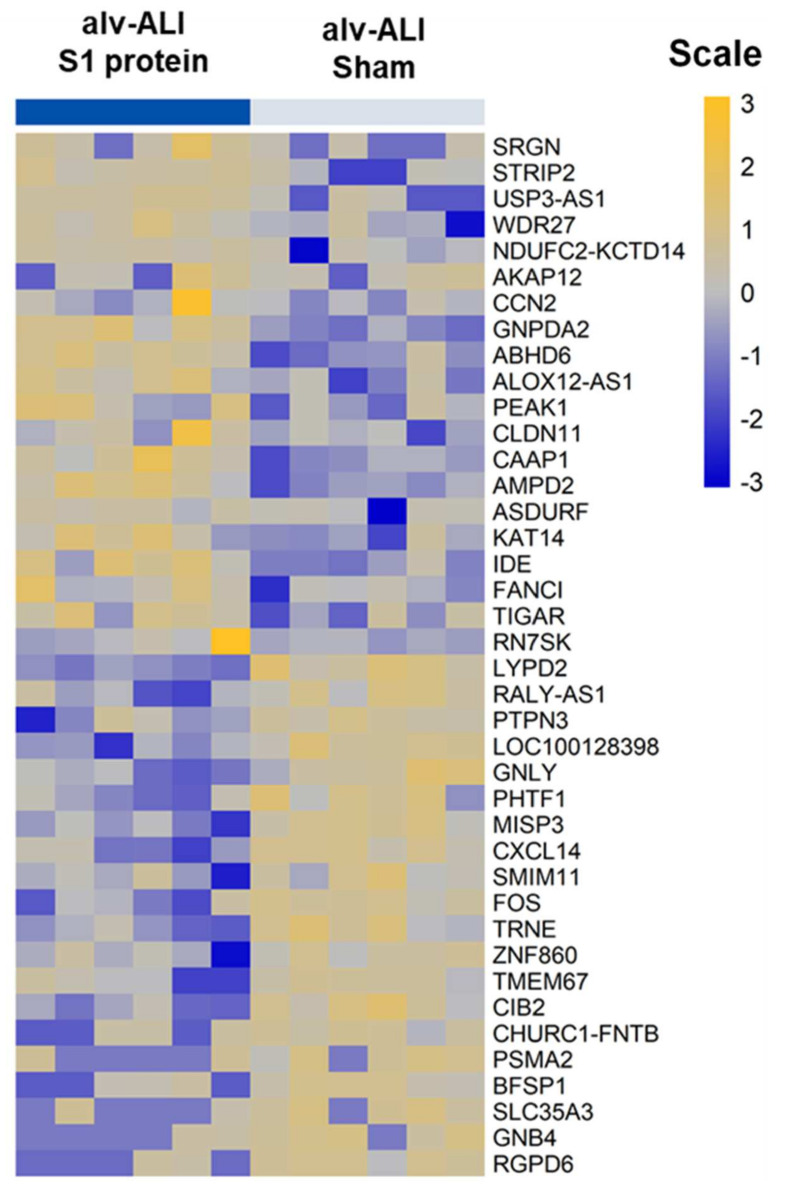


### 3.5. Secreted Cytokines in alv-ALI

**Figure 8:**
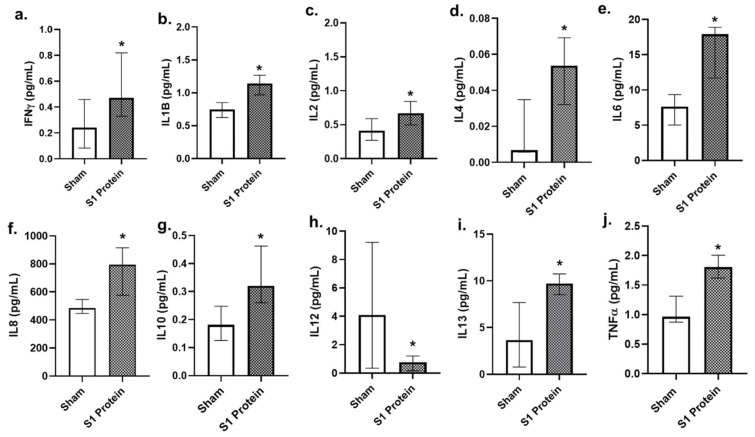
Concentration of secreted proinflammatory cytokines in the basal media of alveolar mucosa model developed at air–liquid interface (alv-ALI). alv-ALI was exposed to 10 nM recombinant SARS-CoV-2 spike glycoprotein S1 (S1 protein) for 24 h and compared to the corresponding sham (**a**–**j**). IFNγ: interferon gamma; IL: interleukin; TNFα: tumor necrosis factor alpha. *n* = 6 per exposure condition; * Significance: *p* < 0.05 (Mann–Whitney *U* test).

Compared to bro-ALI, the significantly altered concentrations of secreted cytokines in alv-ALI at 24 h post SP1 protein exposure compared to the sham (Figure 8) were more pronounced. Concentrations of the proinflammatory cytokines IFNγ, IL1B, IL2, IL4, IL6, IL8, and TNFα (Figure 8a–f and Figure 7i,j) were significantly (*p* < 0.05; *n* = 6) increased, whereas that of IL12 (Figure 8h) was decreased. Consistent with the increased secretion of proinflammatory cytokines, concentration of the anti-inflammatory IL10 (Figure 8g) was also increased in the alv-ALI.

## 4. Discussion

We investigated the effects of SARS-CoV-2 spike glycoprotein 1 on two different lung regions—bronchial and alveolar—using physiologically relevant human lung mucosal models developed at air–liquid interface. Our findings are consistent with other reports [7,8,9] showing increased surface ACE2 expression in both bro-ALI and alv-ALI to S1 protein exposure. Increased protein level expression of ACE2 in bronchial and alveolar tissue has been reported among diabetic patients [6]. Diabetic condition is recognized as an established comorbidity of COVID-19. Surface expression of TLR2 and TLR4 was also increased in both bro-ALI, as well as alv-ALI. SARS-CoV-2 spike glycoprotein has been shown to induce inflammation via TLR2-dependent activation of the NF-kB pathway and consequent release of pro-inflammatory cytokines [28]. Further, SARS-CoV-2 spike glycoprotein is considered to have the strongest protein–protein interaction with TLR4. Activation of TLR4 following binding with SARS-CoV-2 spike glycoprotein is proposed to increase the surface expression of ACE2 facilitating the viral entry [15]. The modelling study further showed that excessive inflammatory and fibrotic responses may occur via TLR4 activation in alveolar cells apart from the activation of interferon signaling, anti-viral, and anti-inflammatory response [15]. Our study revealed a differential response in the bronchial and alveolar regions in response to S1 protein exposure. The bro-ALI model exhibited IFN-γ-mediated anti-viral response whereas the alv-ALI model exhibited a pro-fibrotic response consistent with clinical findings. Therefore, the bro-ALI and alv-ALI mucosa models provide a fast, reliable, and robust screening platform to compare the pathogenicity and immune escape mechanisms of SARS-CoV-2 variants of concern, as well as of other coronaviruses, and to evaluate the long-term consequence of COVID-19 vaccines.

In the bro-ALI, S1 protein exposure induced differential expression of genes primarily involving pathways related to influenza, interferon signaling, antiviral response, and defense response. Moreover, we note that, enrichment of gene ontology terms as specific as coronavirus and COVID-19 are also seen. WNT/ꞵ and sirtuin signaling pathways, both implicated in the pro-fibrotic response of the lung, are also enriched [29,30,31]. In general, growing evidence supports the existence of pro-fibrotic microenvironment in the lung towards the development of pulmonary fibrosis in the long-term [29]. The profibrotic response to S1 protein in the alv-ALI model is highly interesting in light of the evolving knowledge on the development of pulmonary fibrosis as a consequence of severe COVID-19 [18].

Regarding the response to S1 protein in alv-ALI, the enriched gene ontology terms included signaling pathways such as p53, APRIL, tight junction, ILK, and IL1. Each of the mentioned signaling pathways has been associated with fibrosis of the lung. p53 is a tumor suppressor protein involved in a wide range of activities in the lungs, from vascular homeostasis to a protective function in inflammatory reactions. Activation of the p53 pathway induces the senescence of alveolar type II epithelial cells [30]. Anti-viral activities of p53 against influenza is also reported [30]. Elevated serum levels of APRIL, a B-cell-activating factor of the tumor necrosis factor family (BAFF) homolog, was associated with higher incidence of pulmonary fibrosis in a population of patients with systemic sclerosis [31]. The integrin-linked kinase is an intracellular protein shown to be involved in the fibrosis of kidney and liver via the epithelial–mesenchymal transition [32]. Studies in bleomycin-induced pulmonary fibrosis in animal models showed decreased ILK expression during early stages of fibrosis [32]. The IL1 family consists of 11 members including IL1α and IL1ꞵ, which exhibits broad inflammatory activities [33]. Alteration of epithelial barrier function, which is controlled by tight junction proteins, is also associated with IPF [34]. Cytokine storm is a phenomenon of severe COVID-19 leading to acute lung injury and pulmonary fibrosis [35]. The secreted cytokine levels in the alv-ALI model shows a typical pro-inflammatory response via the TLR-mediated signaling pathway [36] in response to S1 protein exposure. Cytokine biology involves highly orchestrated dynamics and cell–cell interaction, particularly in the presence of immune effector cells such as macrophages. In this regard, it is important to note that the bro-ALI and alv-ALI models used herein lack macrophages [37].

A potential limitation of the present study is that the bronchial epithelial cells stem from one donor. In future studies, we aim to collect bronchial tissue from multiple healthy donors, as well as from subjects with predisposed conditions (e.g., asthma, chronic bronchitis, chronic obstructive pulmonary disease) to evaluate molecular responses more precisely. This will however require a high number of experiments.

Increased levels of IL1ꞵ, IL2, IL6, IL8, IL10, and IL12 have been reported in the bronchoalveolar lavage fluid and/or serum of patients with pulmonary fibrosis compared to healthy subjects. Levels of interleukins also varied between patients with different stages of pulmonary fibrosis [38]. In brief, actions of some of the assessed interleukins in the context of pulmonary fibrosis can be summarized as follows: IL1ꞵ (pro-fibrotic), IL4 (pro and anti-fibrotic), IL6 (pro and anti-fibrotic), IL8 (pro-fibrotic), IL10 (anti-fibrotic), IL12 (anti-fibrotic), and IL13 (pro-fibrotic) [38].

The pro-inflammatory and profibrotic action of IL1ꞵ involves recruitment of neutrophils and lymphocytes, and stimulation of fibroblasts to produce collagen and fibrin [39,40,41]. IL4 inhibits T-cell inflammation, induces expression of collagen genes in the fibroblasts, and promotes conversion of fibroblasts to myofibroblasts [42,43]. IL6 exhibits both pro- and anti-inflammatory effects through its actions on fibroblasts and type II pneumocytes, respectively [44,45,46]. IL8 promotes proliferation, differentiation, and migration of mesenchymal progenitor cells in an autocrine manner and induces macrophage migration towards fibrotic foci [47]. The anti-fibrotic effects of IL10 is demonstrated by inhibiting the down regulation of IFNγ [48]. Overexpression of IL10 for longer durations can promote fibrosis by activating the repair M2 macrophages [49]. IL12 overexpression can result in the transformation of Th2 to Th1 cells, thereby inducing IFNγ expression and corresponding inhibition of collagen production [50]. IL13 can induce the expression of α smooth muscle actin and collagen in fibrotic lungs. It also induces the differentiation of fibroblasts to myofibroblasts similar to IL4 [51,52]. Decreased baseline serum IFNγ levels (*p* < 0.01) were reported in COVID-19 patients developing fibrosis (*n* = 46) compared to those not developing fibrosis (*n* = 30) [53]. Increased production of TNFα, together with IL1ꞵ from the macrophages, have been reported in pulmonary fibrosis [40].

## 5. Conclusions

We observed a differential response in the bronchial and alveolar regions after exposure with the SARS-CoV-2 spike glycoprotein protein, the S1 subunit. While the bronchial model exhibited a typical IFN-γ-mediated anti-viral response, the alveolar model exhibited a pro-fibrotic response. Pathway enrichment analysis identified gene ontology terms such as coronavirus and COVID-19, demonstrating the specificity of the response. Since pulmonary fibrosis is a feature and consequence of severe COVID-19, the findings in our lung mucosa models are clinically relevant. It is plausible that the pro-fibrotic environment created due to alteration of p53, APRIL, tight junction, ILK, and IL1 signaling pathways in the alveolar region, together with increased levels of pro-inflammatory cytokines, impacts COVID-19 disease severity. Taken together, the bro-ALI and alv-ALI mucosa models used herein make it possible to perform detailed mechanistic studies using a fast, reliable, and robust screening platform. The models can be further advanced by adding immunocompetent cells like macrophages and by using multiple donors. Studies aimed at understanding the pathogenicity and immune escape mechanisms of SARS-CoV-2 variants of concern, as well as that of other coronaviruses, can be performed using advanced physiologically relevant multicellular lung mucosa models. 

## Figures and Tables

**Table 1:** List of selected enriched pathways in the bronchial and alveolar mucosa models developed at air–liquid interface (bro-ALI and alv-ALI) following exposure to 10 nM recombinant SARS-CoV-2 spike glycoprotein S1 (S1 protein) for 24 h and compared to the corresponding sham. The list of significantly differentially regulated genes used identified in the transcriptomic analysis was used as input (bro-ALI: 117 genes; alv-ALI: 97 genes; Supplementary Tables ST3 and ST4). The canonical pathways listed here were identified using QIAGEN’s Ingenuity Pathway Analysis software (IPA^®^). Significant terms were selected using Fisher’s exact test *p*-values.

Bronchial Mucosa Model (bro-ALI)		
**Canonical Pathway**	***p* Value**	**Molecules**
Role of hypercytokinemia/hyperchemokinemia in the pathogenesis of influenza	2.51 × 10^−12^	*CXCL10, DDX58, EIF2AK2, IFIT2, IFIT3, IRF7, ISG15, MX1, OAS2, RSAD2*
Interferon signaling	1.29 × 10^−10^	*IFI6, IFIT1, IFIT3, IFITM1, IFITM2, ISG15, MX1*
Role of pattern recognition receptors in recognition of bacteria and viruses	4.90 × 10^−4^	*DDX58, EIF2AK2, IFIH1, IRF7, OAS2*
Role of RIG1-like receptors in antiviral innate immunity	9.33 × 10^−4^	*DDX58, IFIH1, IRF7*
WNT/β-catenin signaling	5.89 × 10^−3^	*CSNK2A1, MMP7, PPP2CB, TLE4*
Sirtuin signaling pathway	7.41 × 10^−3^	*H1-3, MAPK7, SCNN1A, TIMM8A, WRN*
Coronavirus replication pathway	1.51 × 10^−2^	*IFITM1, IFITM2*
Role of PKR in interferon induction and antiviral response	1.91 × 10^−2^	*DDX58, EIF2AK2, IFIH1*
**Alveolar Mucosa Model (alv-ALI)**		
**Canonical pathway**	***p* Value**	**Molecules**
p53 signaling	4.07 × 10^−3^	*BBC3, GNL3, TIGAR*
APRIL (a proliferation-inducing ligand)-mediated signaling	8.32 × 10^−3^	*FOS, TNFSF13*
Tight junction signaling	2.04 × 10^−2^	*CLDN11, FOS, MYH9*
Integrin linked kinase signaling	2.69 × 10^−2^	*FOS, MYH9, RSU1*
Agranulocyte adhesion, and diapedesis	3.31 × 10^−2^	*CLDN11, CXCL14, MYH9*
Interleukin-1 signaling	3.80 × 10^−2^	*FOS, GNB4*

## Data Availability

All data presented in the study are available on request from the corresponding authors and are also included in the supplementary section. RNAseq data is deposited at the Gene Expression Omnibus database at NCBI (GSE185657).

## Supplementary Materials

The following are available online at https://www.mdpi.com/article/10.3390/v13122537/s1, Supplementary Figure S1: Assessment of cell viability and cytotoxicity following exposure to recombinant SARS-CoV-2 spike glycoprotein S1 in bronchial and alveolar mucosa model developed at air-liquid interface; Supplementary Figure S2: Surface expression of angiotensin converting enzyme 2, toll-like receptor 2 (TLR2), and TLR4 in bronchial mucosa model developed at air–liquid interface; Supplementary Figure S3: Surface expression of angiotensin converting enzyme 2, toll-like receptor 2 (TLR2), and TLR4 in alveolar mucosa model developed at air–liquid interface; Supplementary Figure S4: Network shapes for IPA canonical pathways; Supplementary Table S1: Detection limit values of cytokines; Supplementary Table S2: Comparison of the fold increase of surface expression of ACE2, TLR2, and TLR4 bro-ALI and alv-ALI; Supplementary Table S3: List of significantly differentially regulated genes in the bronchial mucosa model developed at air–liquid interface; Supplementary Table S4: List of significantly differentially regulated genes in the alveolar mucosa model developed at air–liquid interface.
