# Supplementary material for: Differential Effect of SARS-CoV-2 Spike Glycoprotein 1 on Human Bronchial and Alveolar Lung Mucosa Models: Implications for Pathogenicity
Source: Viruses. 2021 Dec 17;13(12):2537. doi: 10.3390/v13122537 (PMC8708014; doi:10.3390/v13122537)
Supplement: Supplementary file 1 [file viruses-13-02537-s001.zip › viruses-1438869-supplementary.pdf]

viruses

Online Supplementary Material

Transcriptomic analysis workflow:

The "Demultiplex QIAseq UPX 3′ reads" tool of the CLC Genomics Workbench 21.0.1 was used to demultiplex the raw sequencing reads according to the sample indices. The "Quantify QIAseq UPX 3′ workflow" was used to process the demultiplexed sequencing reads with default settings. In short, the reads are annotated with their UMI and are then trimmed for poly(A) and adapter sequences, minimum reads length (15 nucleotides), read quality, and ambiguous nucleotides (maximum of 2). They are then deduplicated using their UMI. Reads are grouped into UMI groups when they (1) start at the same position based on the end of the read to which the UMI is ligated (i.e., Read2 for paired data), (2) are from the same strand, and (3) have identical UMIs. Groups that contain only one read (singletons) are merged into non-singleton groups if the singleton's UMI can be converted to a UMI of a non-singleton group by introducing an SNP (the biggest group is chosen). The reads were then mapped to the Human genome GRCh38. The 'Empirical analysis of DGE' algorithm of the CLC Genomics Workbench 21.0.1 was used for differential expression analysis with default settings. It is an implementation of the 'Exact Test' for two-group comparisons developed by Robinson and Smyth (2008, Small-sample estimation of negative binomial dispersion, with applications to SAGE data) and incorporated in the EdgeR Bioconductor package [1].

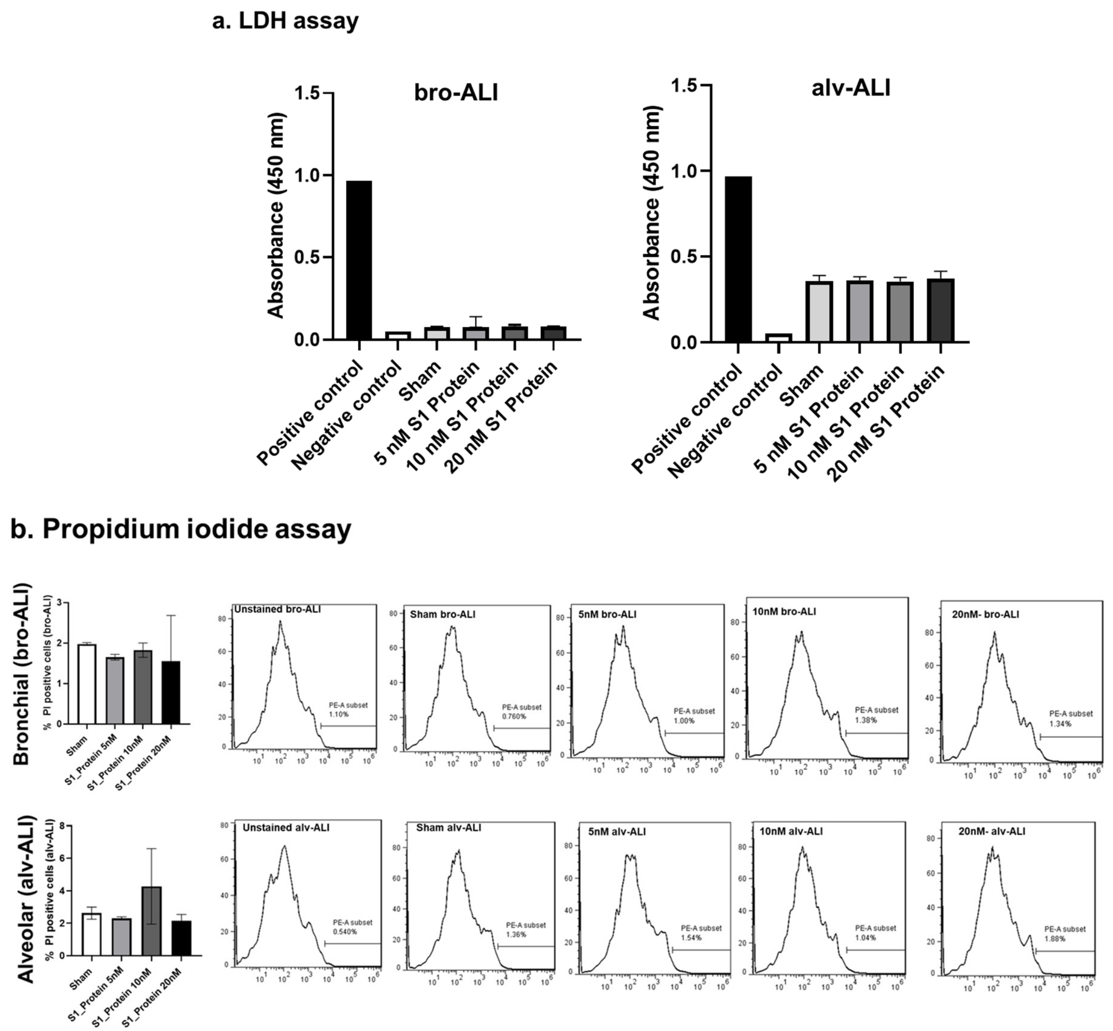
a. LDH assay
bro-ALI
alv-ALI
Absorbance (450 nm)
1.5
1.0
0.5
0.0
Positive control
Negative control
Sham
5 nM S1 Protein
10 nM S1 Protein
20 nM S1 Protein
b. Propidium iodide assay
Bronchial (bro-ALI)
% PI positive cells (bro-ALI)
Alveolar (alv-ALI)
% PI positive cells (alv-ALI)
Sham
S1_Protein 5nM
S1_Protein 10nM
S1_Protein 20nM
Unstained bro-ALI
Sham bro-ALI
5nM bro-ALI
10nM bro-ALI
20nM- bro-ALI
Unstained alv-ALI
Sham alv-ALI
5nM alv-ALI
10nM alv-ALI
20nM- alv-ALI
PE-A subset

Supplementary Figure S1. Cell viability (based on membrane integrity) test was measured by colorimetric lactate dehydrogenase (LDH) assay as well as propidium iodide (PI) staining and flow cytometry.

a. LDH release was measured in the basal media. No significant alteration of cell viability was detected after exposure to recombinant S1 protein (5, 10, 20 nM) in both bronchial and alveolar lung mucosa model developed at the air-liquid interface (bro-ALI and alv-ALI) as shown in bar diagrams (n=3 per exposure condition). Corresponding sham served as control. Kit provided (Thermo Fisher scientific Rockford, lL, US, catalog # 88953) positive control was used for the assay. Cell culture media served as negative control. Data are presented as median and interquartile ranges in bar graphs.

b. Cells were washed with PBS after 24 hours of SARS-CoV-2 spike glycoprotein S1 (S1 protein) exposure and stained with PI solution. After 30 minutes of incubation, cells were assessed by flow cytometry using PE laser. No significant alteration of cell viability was detected after exposure to recombinant S1 protein (5, 10, 20 nM) in both bro-ALI and alv-ALI as shown in bar diagram (n=3 per exposure condition). Representative histograms show gated PI positive cells in percentage from each group. Y axis number of cells and X axis amount of fluorochrome (proportional to number of PI positive cells). Corresponding sham served as control. Data are presented as percentage positive PI cells and interquartile ranges in bar graphs.

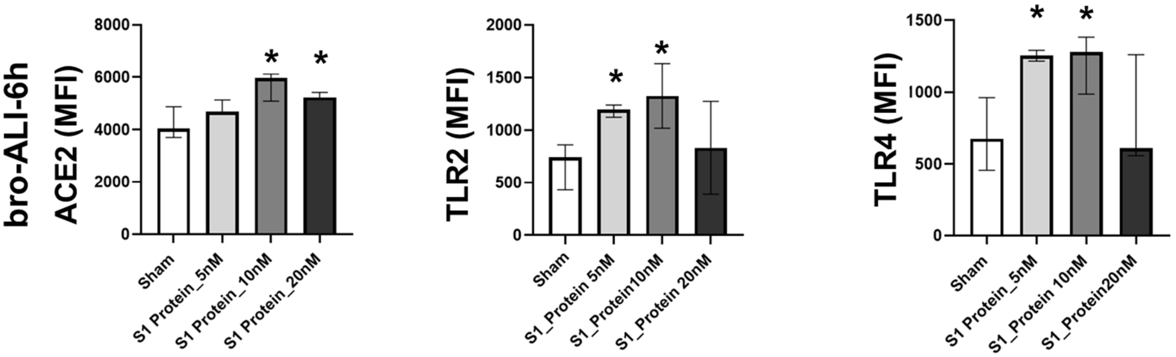

Supplementary Figure S2. Surface expression of angiotensin converting enzyme 2 (ACE2), toll like receptor 2 (TLR2), and TLR4 in bronchial mucosa model developed at air-liquid interface (bro-ALI). Bro-ALI was exposed to recombinant SARS-CoV-2 spike glycoprotein S1 (S1 protein; 5, 10 and 20 nM) for 6 hours (h) and compared to the corresponding sham. ACE2, TLR2, and TLR4 was measured by flow cytometry and data are presented as median fluorescent intensity (MFI) and interquartile ranges. n= 3 per exposure condition; * significance: $p<0.05$ (Mann-Whitney U-test).

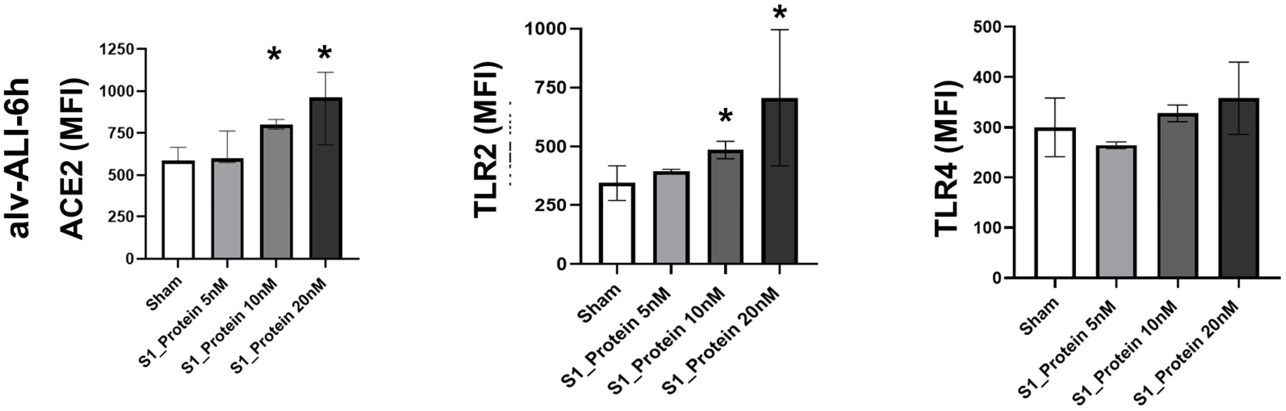

Supplementary Figure S3. Surface expression of angiotensin converting enzyme 2 (ACE2), toll like receptor 2 (TLR2), and TLR4 in alveolar mucosa model developed at air-liquid interface (alv-ALI). alv-ALI was exposed to recombinant SARS-CoV-2 spike glycoprotein S1 (S1 protein; 5, 10 and 20 nM) for 6 hours (h) and compared to the corresponding sham. ACE2, TLR2, and TLR4 was measured by flow cytometry and data are presented as median fluorescent intensity (MFI; interquartile range). n= 3 per exposure condition; * significance: $p<0.05$ (Mann-Whitney U-test).

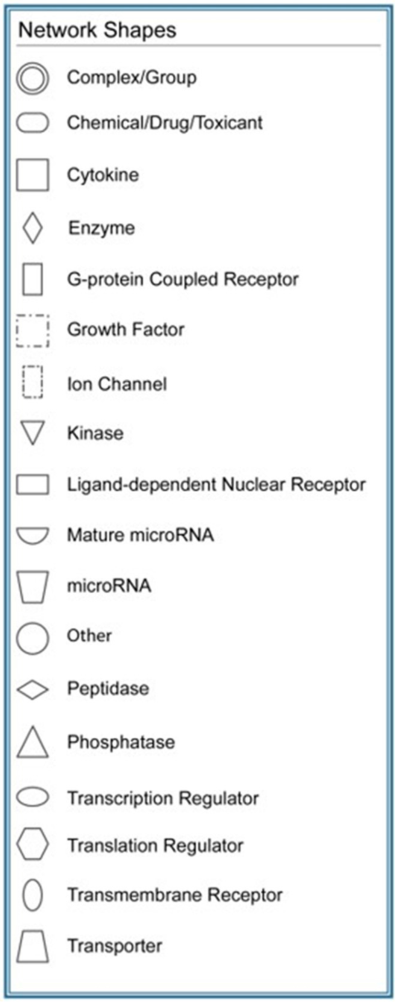

Supplementary Figure S4. Network shapes used in Ingenuity Pathway Analysis.

Supplementary Table S1: Detection limit values of the cytokines. LLOD: Lower limit of detection; ULOQ: Upper limit of quantification. For measuring interleukin (IL) 8, samples were diluted in the ratio of 1:30 and for the MSD Mesoscale analysis samples were diluted in the ratio of 1:2. IFN-γ: Interferon gamma, TNF-α: tumor necrosis factor alpha.

Assay	LLOD pg/mL	ULOQ pg/mL
IFN-γ	0.4665	1560
IL-10	0.0425	346
IL-12p70	0.0780	447
IL-13	0.5225	466
IL-1β	0.1373	583
IL-2	0.0589	1250
IL-4	0.0090	213
IL-6	0.1112	663
IL-8	31.25	2000
TNF-α	0,0494	338

Supplementary Table S2: Comparison of the fold increase of surface expression of angiotensin converting enzyme 2 (ACE2), toll like receptor 2 (TLR2), and TLR4 between the bronchial (bro-ALI) and alveolar (alv-ALI) mucosa model developed at air-liquid interface (ALI). Both bro-ALI and alv-ALI were exposed to 10 nM recombinant SARS-CoV-2 spike glycoprotein S1 (S1 protein) for 6 hour (h) and compared to the corresponding sham. bro-ALI was developed using human primary bronchial epithelial cells and alv-ALI using NCI-H441 (ATCC HTB-174) cell line as representative of human type II pneumocytes. ACE2, TLR2, and TLR4 was measured by flow cytometry. n= 6 per exposure condition; * significance: $p < 0.05$ (Mann-Whitney U-test).

	**bro-ALI** (Fold change)#	**alv-ALI** (Fold change)#	**alv-ALI/bro-ALI**	
			Fold change	p
**ACE2**	1.2*	1.5*	1.3*	0.002
**TLR2**	1.3*	1.4*	1.1	0.48
**TLR4**	1.3*	1.3*	1.0	0.31
#: compared to corresponding sham				

Supplementary Table S3. List of significantly differentially regulated genes (Total: 117; upregulated: 77; down-regulated: 40) in the bronchial mucosa model developed at air-liquid interface (bro-ALI). bro-ALI was exposed to 10 nM recombinant SARS-CoV-2 spike glycoprotein S1 (S1 protein) for 24 hours and compared to sham. n= 6 per exposure condition; significance: p<0.01. An additional filter was applied for expression >0 in at least 5 samples in at least one group.

	Symbol or ID	Fold-change	Description	Ensembl gene ID	Entrez
1.	*ENSG00000278878*	**11.90**		ENSG00000278878	
2.	*EYA1*	**9.76**	EYA transcriptional coactivator and phosphatase 1	ENSG00000104313	2138
3.	*STARD4-AS1*	**5.95**	STARD4 antisense RNA 1	ENSG00000246859	100505678
4.	*CA9*	**5.63**	Carbonic anhydrase 9	ENSG00000107159	768
5.	*ZNF568*	**5.39**	Zinc finger protein 568	ENSG00000198453	374900
6.	*PPP3CB-AS1*	**4.85**	PPP3CB antisense RNA 1 (head to head)	ENSG00000221817	101929145
7.	*TMBIM4*	**4.47**	Transmembrane BAX inhibitor motif containing 4	ENSG00000228144	51643
8.	*RASA4*	**4.46**	RAS p21 protein activator 4	ENSG00000105808	10156
9.	*ENSG00000273489*	**4.28**		ENSG00000273489	
10.	*IQGAP3*	**4.19**	IQ motif containing GTPase activating protein 3	ENSG00000183856	128239
11.	*ADARB1*	**3.89**	Adenosine deaminase RNA specific B1	ENSG00000197381	104
12.	*H1-3*	**3.87**	H1.3 linker histone, cluster member	ENSG00000124575	3007
13.	*ULBP1*	**3.58**	UL16 binding protein 1	ENSG00000111981	80329
14.	*ENSG00000273221*	**3.09**		ENSG00000273221	
15.	*RSAD2*	**2.94**	Radical S-adenosyl methionine domain containing 2	ENSG00000134321	91543
16.	*ENSG00000280614*	**2.76**		ENSG00000280614	
17.	*ENSG00000250135*	**2.70**		ENSG00000250135	
18.	*ENSG00000280800*	**2.67**		ENSG00000280800	
19.	*B3GNT4*	**2.65**	UDP-GlcNAc:betaGal beta-1,3-N-acetylglucosaminyltransferase 4	ENSG00000176383	79369
20.	*CXCL10*	**2.62**	C-X-C motif chemokine ligand 10	ENSG00000169245	3627
21.	*TLCD5*	**2.56**	TLC domain containing 5	ENSG00000181264	219902
22.	*VEGFC*	**2.55**	Vascular endothelial growth factor C	ENSG00000150630	7424
23.	*MX2*	**2.50**	MX dynamin like GTPase 2	ENSG00000183486	4600
24.	*ENSG00000281181*	**2.50**		ENSG00000281181	
25.	*ENSG00000269973*	**2.49**		ENSG00000269973	
26.	*ENSG00000282034*	**2.45**		ENSG00000282034	
27.	*IFIT1*	**2.37**	Interferon induced protein with tetratricopeptide repeats 1	ENSG00000185745	3434
28.	*PINK1-AS*	**2.33**	PINK1 antisense RNA	ENSG00000117242	100861548
29.	*IFIT3*	**2.31**	Interferon induced protein with tetratricopeptide repeats 3	ENSG00000119917	3437
30.	*LINC01006*	**2.28**	Long intergenic non-protein coding RNA 1006	ENSG00000182648	100506380
31.	*ARHGAP23*	**2.19**	Rho GTPase activating protein 23	ENSG00000275832	57636
32.	*IFI6*	**2.16**	Interferon alpha inducible protein 6	ENSG00000126709	2537

33.	*PRELID2*	**2.14**	PRELI domain containing 2	ENSG00000186314	153768
34.	*IFIT2*	**2.14**	Interferon induced protein with tetratricopeptide repeats 2	ENSG00000119922	3433
35.	*MLKL*	**2.09**	Mixed lineage kinase domain like pseudokinase	ENSG00000168404	197259
36.	*CTHRC1*	**2.09**	Collagen triple helix repeat containing 1	ENSG00000164932	115908
37.	*IFI44L*	**1.99**	Interferon induced protein 44 like	ENSG00000137959	10964
38.	*AUTS2*	**1.91**	Activator of transcription and developmental regulator AUTS2	ENSG00000158321	26053
39.	*CASD1*	**1.88**	CAS1 domain containing 1	ENSG00000127995	64921
40.	*MX1*	**1.84**	MX dynamin like GTPase 1	ENSG00000157601	4599
41.	*HPS3*	**1.84**	HPS3 biogenesis of lysosomal organelles complex 2 subunit 1	ENSG00000163755	84343
42.	*OAS2*	**1.84**	2'-5'-oligoadenylate synthetase 2	ENSG00000111335	4939
43.	*ENSG00000225963*	**1.82**		ENSG00000225963	
44.	*DDX10*	**1.79**	DEAD-box helicase 10	ENSG00000178105	1662
45.	*IFIT5*	**1.77**	Interferon induced protein with tetratricopeptide repeats 5	ENSG00000152778	24138
46.	*KLK7*	**1.74**	Kallikrein related peptidase 7	ENSG00000169035	5650
47.	*TXLNG*	**1.74**	Taxilin gamma	ENSG00000086712	55787
48.	*MAPK7*	**1.69**	Mitogen-activated protein kinase 7	ENSG00000166484	5598
49.	*DDX58*	**1.69**	DExD/H-box helicase 58	ENSG00000107201	23586
50.	*CORO1C*	**1.61**	coronin 1C	ENSG00000110880	23603
51.	*APOBEC3B*	**1.61**	Apolipoprotein B mRNA editing enzyme catalytic subunit 3B	ENSG00000179750	9582
52.	*XAF1*	**1.60**	XIAP associated factor 1	ENSG00000132530	54739
53.	*SP110*	**1.60**	SP110 nuclear body protein	ENSG00000135899	3431
54.	*ISG15*	**1.60**	ISG15 ubiquitin like modifier	ENSG00000187608	9636
55.	*IFIH1*	**1.59**	Interferon induced with helicase C domain 1	ENSG00000115267	64135
56.	*PTPN13*	**1.56**	Protein tyrosine phosphatase non-receptor type 13	ENSG00000163629	5783
57.	*SAMD4A*	**1.56**	Sterile alpha motif domain containing 4A	ENSG00000020577	23034
58.	*GBP1*	**1.52**	Guanylate binding protein 1	ENSG00000117228	2633
59.	*PPM1K*	**1.50**	Protein phosphatase, Mg2+/Mn2+ dependent 1K	ENSG00000163644	152926
60.	*CWC22*	**1.50**	CWC22 spliceosome associated protein homolog	ENSG00000163510	57703
61.	*OMA1*	**1.48**	OMA1 zinc metallopeptidase	ENSG00000162600	115209
62.	*QPRT*	**1.46**	Quinolinate phosphoribosyltransferase	ENSG00000103485	23475
63.	*SAMD9*	**1.45**	Sterile alpha motif domain containing 9	ENSG00000205413	54809
64.	*PPP2CB*	**1.45**	Protein phosphatase 2 catalytic subunit beta	ENSG00000104695	5516
65.	*TLE4*	**1.44**	TLE family member 4, transcriptional corepressor	ENSG00000106829	7091
66.	*NT5C3A*	**1.44**	5'-nucleotidase, cytosolic IIIA	ENSG00000122643	51251

67.	*IRF7*	**1.39**	Interferon regulatory factor 7	ENSG00000185507	3665
68.	*DEK*	**1.39**	DEK proto-oncogene	ENSG00000124795	7913
69.	*CMIP*	**1.39**	c-Maf inducing protein	ENSG00000153815	80790
70.	*IFI27*	**1.37**	Interferon alpha inducible protein 27	ENSG00000165949	3429
71.	*PNPT1*	**1.36**	Polyribonucleotide nucleotidyltransfer-ase 1	ENSG00000138035	87178
72.	*IFITM1*	**1.34**	Interferon induced transmembrane protein 1	ENSG00000185885	8519
73.	*UCK2*	**1.34**	Uridine-cytidine kinase 2	ENSG00000143179	7371
74.	*CAPS*	**1.33**	Calcyphosine	ENSG00000105519	828
75.	*EIF2AK2*	**1.32**	Eukaryotic translation initiation factor 2 alpha kinase 2	ENSG00000055332	5610
76.	*UBL3*	**1.29**	Ubiquitin like 3	ENSG00000122042	5412
77.	*IFITM2*	**1.28**	Interferon induced transmembrane protein 2	ENSG00000185201	10581
78.	*CSNK2A1*	**-1.22**	Casein kinase 2 alpha 1	ENSG00000101266	1457
79.	*ATP6V1D*	**-1.26**	ATPase H+ transporting V1 subunit D	ENSG00000100554	51382
80.	*MRPL46*	**-1.28**	Mitochondrial ribosomal protein L46	ENSG00000259494	26589
81.	*TWF2*	**-1.30**	Twinfilin actin binding protein 2	ENSG00000247596	11344
82.	*SCNN1A*	**-1.32**	Sodium channel epithelial 1 subunit al-pha	ENSG00000111319	6337
83.	*MMP7*	**-1.35**	Matrix metallopeptidase 7	ENSG00000137673	4316
84.	*ANKRD39*	**-1.37**	Ankyrin repeat domain 39	ENSG00000213337	51239
85.	*NGDN*	**-1.43**	Neuroguidin	ENSG00000129460	25983
86.	*SYNRG*	**-1.44**	Synergin gamma	ENSG00000275066	11276
87.	*TDP2*	**-1.44**	Tyrosyl-DNA phosphodiesterase 2	ENSG00000111802	51567
88.	*BCAS2*	**-1.45**	BCAS2 pre-mRNA processing factor	ENSG00000116752	10286
89.	*TIMM8A*	**-1.45**	Translocase of inner mitochondrial membrane 8A	ENSG00000126953	1678
90.	*PDPK1*	**-1.45**	3-phosphoinositide dependent protein kinase 1	ENSG00000140992	5170
91.	*ATP9A*	**-1.46**	ATPase phospholipid transporting 9A (putative)	ENSG00000054793	10079
92.	*BET1*	**-1.48**	Bet1 golgi vesicular membrane traf-ficking protein	ENSG00000105829	10282
93.	*PREPL*	**-1.54**	Prolyl endopeptidase like	ENSG00000138078	9581
94.	*CDK10*	**-1.57**	Cyclin dependent kinase 10	ENSG00000185324	8558
95.	*TPCN1*	**-1.62**	Two pore segment channel 1	ENSG00000186815	53373
96.	*FAHD2B*	**-1.67**	Fumarylacetoacetate hydrolase do-main containing 2B	ENSG00000144199	151313
97.	*SPOUT1*	**-1.68**	SPOUT domain containing methyl-transferase 1	ENSG00000198917	51490
98.	*ASCC1*	**-1.75**	Activating signal cointegrator 1 com-plex subunit 1	ENSG00000138303	51008
99.	*PNMA1*	**-1.75**	PNMA family member 1	ENSG00000176903	9240
100.	*WRN*	**-1.77**	WRN RecQ like helicase	ENSG00000165392	7486
101.	*MOAP1*	**-1.83**	Modulator of apoptosis 1	ENSG00000165943	64112
102.	*MMAA*	**-1.96**	Metabolism of cobalamin associated A	ENSG00000151611	166785
103.	*FAM153CP*	**-2.06**	Protein FAM153C	ENSG00000204677	653316

104.	*ANKZF1*	**-2.11**	Ankyrin repeat and zinc finger peptidyl tRNA hydrolase 1	ENSG00000163516	55139
105.	*INTS9*	**-2.15**	Integrator complex subunit 9	ENSG00000104299	55756
106.	*DPYD*	**-2.20**	Dihydropyrimidine dehydrogenase	ENSG00000188641	1806
107.	*RIC3*	**-2.36**	RIC3 acetylcholine receptor chaperone	ENSG00000166405	79608
108.	*TMEM51-AS1*	**-2.85**	TMEM51 antisense RNA 1	ENSG00000175147	200197
109.	*ENSG00000184809*	**-3.19**		ENSG00000184809	
110.	*SNAI3-AS1*	**-3.23**	SNAI3 antisense RNA 1	ENSG00000260630	197187
111.	*JRK*	**-3.25**	Jrk helix-turn-helix protein	ENSG00000234616	8629
112.	*ENSG00000260669*	**-3.41**		ENSG00000260669	
113.	*MAP4K2*	**-3.52**	Mitogen-activated protein kinase kinase kinase kinase 2	ENSG00000168067	5871
114.	*GOLGA6L4*	**-4.20**	Golgin A6 family like 4	ENSG00000184206	643707
115.	*CES4A*	**-4.55**	Carboxylesterase 4A	ENSG00000172824	283848
116.	*POLR2J4*	**-5.86**	RNA polymerase II subunit J4, pseudogene	ENSG00000214783	84820
117.	*WDR27*	**-7.64**	WD repeat domain 27	ENSG00000184465	253769

Supplementary Table S4. List of significantly differentially regulated genes (Total: 97; upregulated: 47; down-regulated: 50) in the alveolar mucosa model developed at air-liquid interface (alv-ALI). alv-ALI was exposed to 10nM recombinant SARS-CoV-2 spike glycoprotein S1 (S1 protein) for 24 hours and compared to sham. n= 6 per exposure condition; significance: p<0.01. An additional filter was applied for expression >0 in at least 5 samples in at least one group.

	**Symbol or ID**	**Fold-change**	**Description**	**Ensembl gene ID**	**Entrez**
1.	*ENSG00000256825*	**26.79**		ENSG00000256825	
2.	*SRGN*	**15.01**	Serglycin	ENSG00000122862	5552
3.	*STRIP2*	**4.13**	Striatin interacting protein 2	ENSG00000128578	57464
4.	*USP3-AS1*	**3.83**	USP3 antisense RNA 1	ENSG00000259248	100130855
5.	*WDR27*	**3.42**	WD repeat domain 27	ENSG00000184465	253769
6.	*NDUFC2-KCTD14*	**3.33**	NDUFC2-KCTD14 readthrough	ENSG00000259112	100532726
7.	*AKAP12*	**3.13**	A-kinase anchoring protein 12	ENSG00000131016	9590
8.	*CCN2*	**3.11**	Cellular communication network factor 2	ENSG00000118523	1490
9.	*GNPDA2*	**3.02**	Glucosamine-6-phosphate deaminase 2	ENSG00000163281	132789
10.	*ABHD6*	**2.96**	Abhydrolase domain containing 6, acylglycerol lipase	ENSG00000163686	57406
11.	*ALOX12-AS1*	**2.45**	ALOX12 antisense RNA 1	ENSG00000215067	100506713
12.	*PEAK1*	**2.42**	Pseudopodium enriched atypical kinase 1	ENSG00000173517	79834
13.	*ENSG00000263731*	**2.36**		ENSG00000263731	
14.	*CLDN11*	**2.20**	Claudin 11	ENSG00000013297	5010
15.	*CAAP1*	**2.01**	Caspase activity and apoptosis inhibitor 1	ENSG00000120159	79886
16.	*AMPD2*	**2.00**	Adenosine monophosphate deaminase 2	ENSG00000116337	271

17.	*ASDURF*	**1.94**	ASNSD1 upstream open reading frame	ENSG00000286053	110599588
18.	*KAT14*	**1.92**	Lysine acetyltransferase 14	ENSG00000149474	57325
19.	*IDE*	**1.85**	Insulin degrading enzyme	ENSG00000119912	3416
20.	*FANCI*	**1.84**	FA complementation group I	ENSG00000140525	55215
21.	*TIGAR*	**1.83**	TP53 induced glycolysis regulatory phosphatase	ENSG00000078237	57103
22.	*RN7SK*	**1.79**	RNA component of 7SK nuclear ribonucleoprotein	ENSG00000202198	125050
23.	*CEP78*	**1.79**	Centrosomal protein 78	ENSG00000148019	84131
24.	*GLIPR1*	**1.78**	GLI pathogenesis related 1	ENSG00000139278	11010
25.	*CCDC24*	**1.71**	Coiled-coil domain containing 24	ENSG00000159214	149473
26.	*HELLS*	**1.70**	Helicase, lymphoid specific	ENSG00000119969	3070
27.	*GNL3*	**1.69**	G protein nucleolar 3	ENSG00000163938	26354
28.	*CC2D1B*	**1.67**	Coiled-coil and C2 domain containing 1B	ENSG00000154222	200014
29.	*NIPAL1*	**1.59**	NIPA like domain containing 1	ENSG00000163293	152519
30.	*CALCOCO1*	**1.57**	Calcium binding and coiled-coil domain 1	ENSG00000012822	57658
31.	*ADO*	**1.53**	2-aminoethanethiol dioxygenase	ENSG00000181915	84890
32.	*THAP3*	**1.53**	THAP domain containing 3	ENSG00000041988	90326
33.	*GON4L*	**1.49**	Gon-4 like	ENSG00000116580	54856
34.	*HERC4*	**1.48**	HECT and RLD domain containing E3 ubiquitin protein ligase 4	ENSG00000148634	26091
35.	*MEN1*	**1.46**	Menin 1	ENSG00000133895	4221
36.	*ACBD6*	**1.46**	Acyl-CoA binding domain containing 6	ENSG00000230124	84320
37.	*TMX2*	**1.44**	Thioredoxin related transmembrane protein 2	ENSG00000213593	51075
38.	*DNMT1*	**1.44**	DNA methyltransferase 1	ENSG00000130816	1786
39.	*NBN*	**1.43**	Nibrin	ENSG00000104320	4683
40.	*POLR2M*	**1.43**	RNA polymerase II subunit M	ENSG00000255529	81488
41.	*PNN*	**1.42**	Pinin, desmosome associated protein	ENSG00000100941	5411
42.	*FBXO33*	**1.42**	F-box protein 33	ENSG00000165355	254170
43.	*CDK11A*	**1.39**	Cyclin dependent kinase 11A	ENSG00000008128	728642
44.	*TMEM131*	**1.39**	Transmembrane protein 131	ENSG00000075568	23505
45.	*CHD1L*	**1.35**	Chromodomain helicase DNA binding protein 1 like	ENSG00000131778	9557
46.	*ATL2*	**1.34**	atlastin GTPase 2	ENSG00000119787	64225
47.	*RSU1*	**1.33**	Ras suppressor protein 1	ENSG00000148484	6251
48.	*PLEKHB2*	**-1.25**	Pleckstrin homology domain containing B2	ENSG00000115762	55041
49.	*PKN1*	**-1.31**	Protein kinase N1	ENSG00000123143	5585
50.	*BBC3*	**-1.34**	BCL2 binding component 3	ENSG00000105327	27113
51.	*MYH9*	**-1.36**	Myosin heavy chain 9	ENSG00000100345	4627
52.	*MRM1*	**-1.39**	Mitochondrial rRNA methyltransferase 1	ENSG00000278619	79922
53.	*TMEM181*	**-1.40**	Transmembrane protein 181	ENSG00000146433	57583

54.	*RNF141*	**-1.42**	Ring finger protein 141	ENSG00000110315	50862
55.	*AKR7A2*	**-1.43**	Aldo-keto reductase family 7 member A2	ENSG00000053371	8574
56.	*RCOR3*	**-1.47**	REST corepressor 3	ENSG00000117625	55758
57.	*OTULINL*	**-1.50**	OTU deubiquitinase with linear linkage specificity like	ENSG00000145569	54491
58.	*LHFPL2*	**-1,50**	LHFPL tetraspan subfamily member 2	ENSG00000145685	10184
59.	*GARRE1*	**-1.51**	Granule associated Rac and RHOG effector 1	ENSG00000166398	9710
60.	*NNMT*	**-1.52**	Nicotinamide N-methyltransferase	ENSG00000166741	4837
61.	*C19orf12*	**-1.53**	Chromosome 19 open reading frame 12	ENSG00000131943	83636
62.	*ENSG00000273272*	**-1.54**		ENSG00000273272	
63.	*ENSG00000283103*	**-1.54**		ENSG00000283103	
64.	*TCP11L2*	**-1.56**	T-complex 11 like 2	ENSG00000166046	255394
65.	*VPS45*	**-1.62**	Vacuolar protein sorting 45 homolog	ENSG00000136631	11311
66.	*TNFSF13*	**-1.71**	TNF superfamily member 13	ENSG00000161955	8741
67.	*ZNF12*	**-1.73**	Zinc finger protein 12	ENSG00000164631	7559
68.	*ACOX3*	**-1.76**	Acyl-CoA oxidase 3, pristanoyl	ENSG00000087008	8310
69.	*LYPD2*	**-1.81**	LY6/PLAUR domain containing 2	ENSG00000197353	137797
70.	*RALY-AS1*	**-1.86**	RALY antisense RNA 1	ENSG00000285230	101926888
71.	*PTPN3*	**-1.86**	Protein tyrosine phosphatase non-receptor type 3	ENSG00000070159	5774
72.	*LOC100128398*	**-1,88**	Uncharacterized LOC100128398	ENSG00000176593	100128398
73.	*GNLY*	**-1.99**	Granulysin	ENSG00000115523	10578
74.	*PHTF1*	**-2.05**	Putative homeodomain transcription factor 1	ENSG00000116793	10745
75.	*ENSG00000272667*	**-2.07**		ENSG00000272667	
76.	*MISP3*	**-2.12**	MISP family member 3	ENSG00000141854	113230
77.	*CXCL14*	**-2.13**	C-X-C motif chemokine ligand 14	ENSG00000145824	9547
78.	*SMIM11*	**-2.17**	Small integral membrane protein 11	ENSG00000205670	54065
79.	*FOS*	**-2.18**	Fos proto-oncogene, AP-1 transcription factor subunit	ENSG00000170345	2353
80.	*ENSG00000250903*	**-2.24**		ENSG00000250903	
81.	*TRNE*	**-2.53**	tRNA	ENSG00000210194	4556
82.	*ZNF860*	**-2.88**	Zinc finger protein 860	ENSG00000197385	344787
83.	*TMEM67*	**-2.99**	Transmembrane protein 67	ENSG00000164953	91147
84.	*ENSG00000272279*	**-3.03**		ENSG00000272279	
85.	*CIB2*	**-3.06**	Calcium and integrin binding family member 2	ENSG00000136425	10518
86.	*CHURC1-FNTB*	**-3.17**	CHURC1-FNTB readthrough	ENSG00000125954	100529261
87.	*ENSG00000258311*	**-3.27**		ENSG00000258311	
88.	*ENSG00000255121*	**-3.53**		ENSG00000255121	
89.	*PSMA2*	**-4.11**	Proteasome 20S subunit alpha 2	ENSG00000256646	5683
90.	*ENSG00000255641*	**-4.16**		ENSG00000255641	

91.	*BFSP1*	**-4.33**	Beaded filament structural protein 1	ENSG00000125864	631
92.	*ENSG00000254929*	**-4.90**		ENSG00000254929	
93.	*SLC35A3*	**-5.70**	Solute carrier family 35 member A3	ENSG00000283761	23443
94.	*GNB4*	**-5.99**	G protein subunit beta 4	ENSG00000114450	59345
95.	*ENSG00000274204*	**-5.99**		ENSG00000274204	
96.	*RGPD6*	**-7.51**	RANBP2 like and GRIP domain containing 6	ENSG00000183054	729540
97.	*ENSG00000265257*	**-10.61**		ENSG00000265257	

Reference:

1. 1.Robinson, M. D. M., D.J.; Smyth, G.K. , edgeR: a Bioconductor package for differential expression analysis of digital gene expression data. *Bioinformatics* **2010,** *26(1):* (1), pp139-40.
